# Acupuncture for Atraumatic Shoulder Conditions: Protocol for a Systematic Review and Meta-Analysis

**DOI:** 10.3389/fmed.2022.720551

**Published:** 2022-03-07

**Authors:** Yi Wang, Yan Xu, Yu Peng, Shichuan Liao, Guogang Dai, Tao Li

**Affiliations:** ^1^Cervicodynia/Omalgia/Lumbago/Sciatica Department 2, Sichuan Province Orthopedic Hospital, Chengdu, China; ^2^Experiment Teaching Center for Preclinical Medicine, Chengdu Medical College, Chengdu, China; ^3^School of Sports Medicine and Health, Postgraduate School, Chengdu Sport Institute, Chengdu, China

**Keywords:** acupuncture, atraumatic shoulder conditions, protocol, systematic review, meta-analysis

## Abstract

**Introduction:**

Shoulder pain is one of the most common musculoskeletal disorders among adults and is caused by a variety of shoulder conditions. The popularity of different acupuncture methods in the nonsurgical treatment of shoulder pain has recently increased. However, evidence regarding the efficacy of acupuncture for shoulder pain is inconsistent, and there is a lack of supporting evidence regarding the overall efficacy of different acupuncture methods for shoulder pain.

**Methods and Analysis:**

A systematic review will be conducted to assess the effectiveness of a wide range of acupuncture techniques for atraumatic shoulder conditions. The PubMed, EMBASE, Cochrane Central Register of Controlled Trials, Web of Science, Ovid MEDLINE, Chinese Biomedical Literature, Chinese National Knowledge Infrastructure, Wanfang, and Chongqing VIP databases will be searched to identify eligible studies. Studies will be selected according to preset inclusion and exclusion criteria and relevant data will be extracted from the final included studies. The heterogeneity, risk of bias, publication bias and evidence quality of the studies will be assessed, and a subgroup analysis and sensitivity analysis will be performed.

**Systematic Review Registration:**

PROSPERO registration number CRD42021249625.

## Introduction

Shoulder pain is considered one of the most common causes of musculoskeletal problems in primary care, with a prevalence of 16 to 26% in the general population and a lifetime prevalence of 7 to 67% (1, 2). It often results in weakness and dysfunction of the upper extremity, compromising an individual's health status (3, 4). The continuous optimization of strategies for treating shoulder pain remains an ongoing clinical challenge.

The shoulder joint consists of 4 joints, the glenohumeral joint, sternoclavicular joint, acromioclavicular joint, and scapulothoracic articulation, and the capsules, ligaments, tendons and muscles that are attached to these joints (5). The complexity of the structure leads to the complexity of shoulder pain-related pathology. Atraumatic shoulder pain is usually related to a variety of shoulder conditions, including rotator cuff tears, subacromial impingement, osteoarthritis, adhesive capsulitis, tendinitis, tendinopathy, tenosynovitis, and bursitis (6–10). Because of the diversity of the pathology causing shoulder pain, the diagnosis of shoulder pain varies in many situations (7). First, the results of a physical examination of the shoulder are affected by a certain subjectivity of the examiner (9). The sensitivity and specificity of clinical shoulder tests have been reported to fluctuate over a range from 0.21 to 0.82 and 0.53 to 0.98 (11–13). The interobserver agreement of the diagnosis of shoulder pain was reported to be only 60% (9). These characteristics make it difficult to accurately distinguish different pathologies and diagnoses of shoulder pain. Although diagnostic imaging, including ultrasounds, radiograms, arthrograms, computed tomography and magnetic resonance imaging, can help to assess shoulder pain, each of these imaging methods has its own disadvantages in evaluating shoulder conditions (14–16). Thus, all studies on specific shoulder conditions may be biassed due to an inaccurate diagnosis during the initial inclusion stage, and the conclusions of these studies are usually about shoulder conditions as a whole, rather than specific described conditions. In view of the inaccuracy and inconsistency of the abovementioned diagnoses of shoulder pain, we propose that these conditions be considered as a whole when assessing the effectiveness of the methods used for the management of shoulder pain.

Nonsurgical treatment of shoulder conditions includes manual therapy, physical therapy, nonsteroidal anti-inflammatory drugs, analgesic agents, platelet-rich plasma injections and glucocorticoid injections (17–21). Some of these methods are supported by low- and moderate-quality evidence for the treatment of a specific shoulder condition, while evidence supporting other methods is inconclusive (17, 22, 23). Acupuncture is a widely used complementary and alternative treatment for shoulder pain (24–29). However, these studies have inconsistent conclusions about the efficacy of acupuncture treatment for shoulder pain. One reason may be the wide variety of acupuncture techniques in clinical use, including acupuncture, electroacupuncture, elongated needle acupuncture, internal heat-type acupuncture, intradermal needle embedding therapy, warm acupuncture, fire needle acupuncture, auricular acupuncture, abdominal acupuncture, acupoint injection and floating needle therapy. All existing reports on the efficacy of acupuncture for shoulder conditions focused on one or only a few acupuncture techniques, and conclusions about “acupuncture” based on only a few techniques are not sufficient. A previous review in 2005 failed to conclude whether acupuncture is effective or harmful for shoulder conditions (30). A review in 2010 found that acupuncture was not more effective than a placebo or ultrasound treatment (21). A review in 2018 showed very low-quality evidence to support the use of trigger point dry needling in the shoulder region for treating patients with upper extremity pain or dysfunction (31). Another review in 2020 showed very little evidence that acupuncture has a superior effect on adhesive capsulitis when compared to other interventions (32). However, there are fundamental differences in the pathogenesis between traumatic and nontraumatic shoulder pain, and most existing reviews did not distinguish between the two causes of shoulder pain in participants, potentially leading to bias in the conclusions. In recent decades, research on acupuncture for shoulder pain has continued, with new methods of acupuncture being developed and new evidence being produced that acupuncture is superior to many other treatments in relieving shoulder pain (33–40). Thus, it is necessary to revisit the overall comparison between acupuncture and other interventions for common atraumatic shoulder pain. The aim of the present study was to systematically review and assess the effectiveness of a wide range of acupuncture techniques for common atraumatic shoulder conditions.

## Methods and Analysis

This systematic review and meta-analysis protocol will be conducted according to the Preferred Reporting Items for Systematic Reviews and Meta-Analysis Protocols (PRISMA-P) statement (41). This review protocol has been registered on PROSPERO with registration number CRD42021249625.

### Inclusion Criteria

#### Types of Studies

Only randomised controlled clinical trials will be included.

#### Types of Participants

This review will include patients who are older than 18 years of age with atraumatic shoulder conditions that have been nonsurgically treated. The following studies will be excluded: studies among populations with “red flag” diagnoses (e.g., rheumatoid arthritis, tuberculosis, cancer, infection, tumour); studies among populations with rotator cuff tears from major trauma (e.g., a sports injury, car accident, fall from a height that exceeds one's own height) or other major trauma-related conditions; and studies among populations with glenoid labrum pathologies, previous surgery on the affected shoulder, neck disorders, multisite musculoskeletal pain, relevant systemic diseases and disorders, or neurological disorders.

#### Types of Interventions

Any type of acupuncture will be included, such as acupuncture, electroacupuncture, elongated needle acupuncture, internal heat-type acupuncture, intradermal needle-embedding therapy, warm acupuncture, fire needle acupuncture, auricular acupuncture, abdominal acupuncture, acupoint injection and floating needle therapy.

#### Types of Comparators

Control interventions may include no treatment, sham acupuncture (acupuncture at a point that is not an acupuncture point), physical therapy, nonsteroidal anti-inflammatory drugs, analgesic agents, and the local injection of steroids and/or local anaesthetics.

#### Types of Outcome Measures

The primary outcome will be shoulder pain intensity, measured by the visual analogue scale (VAS) or numeric rating scale (NRS). The results will be expressed as percentages to describe the distributions and 95% CIs to measure the reliability. The χ^2^ test and I^2^ statistic will be applied to evaluate the heterogeneity of the studies. In addition, 95% CIs will be used to measure the reliability. Means and SDs will be calculated to describe the continuous variables.

The secondary outcomes will include shoulder function, measured by validated scales, such as shoulder range of motion (ROM) assessments, the Simple Shoulder Test (SST), Constant-Murley score (CMS), Shoulder Pain and Disability Index (SPADI) total scale, Dutch Shoulder Disability Questionnaire (SDQ-NL), and University of California-Los Angeles (UCLA) Shoulder rating scale; health-related quality of life, measured by validated scales, such as the Short-Form 36 (SF-36) Health Survey or EuroQoL EQ-5D; and the occurrence of adverse events. The results will be expressed as percentages to describe the distributions and 95% CIs to measure the reliability. The χ^2^ test and I^2^ statistic were applied to evaluate the heterogeneity of the studies. In addition, the 95% CIs will be used to measure the reliability. Means and SDs will be calculated to describe continuous variables.

### Search Strategy

The following databases will be searched from January 2000 until May 1, 2021: PubMed, EMBASE, the Cochrane Central Register of Controlled Trials, Web of Science, Ovid MEDLINE, the Chinese Biomedical Literature Database, the Chinese National Knowledge Infrastructure database, the Wanfang database, and the Chongqing VIP database. The reference lists of eligible trials and previous reviews will be hand-checked for eligible studies. There will be no restriction on language. Information from articles that are not in the English or Chinese will be translated using Google's translation tools to acquire relevant information when available. An example search strategy for PubMed is shown in Table 1.

**Table 1:** Search strategy for PubMed.

**Search**	**Query**
#1	“shoulder pain” [Title/Abstract] OR “shoulder conditions” [Title/Abstract] OR “rotator cuff” [Title/Abstract] OR “subacromial impingement” [Title/Abstract] OR “shoulder impingement syndrome” [Title/Abstract] OR “adhesive capsulitis” [Title/Abstract] OR “frozen shoulder” [Title/Abstract] OR “tendinopathy” [Title/Abstract] OR “tendinitis” [Title/Abstract] OR “tenosynovitis” [Title/Abstract] OR “bursitis” [Title/Abstract] OR “osteoarthritis” [Title/Abstract]
#2	“acupuncture” [Title/Abstract] OR “electroacupuncture” [Title/Abstract] OR “intradermal needle embedding” [Title/Abstract] OR “acupoint” [Title/Abstract] OR “floating needle” [Title/Abstract]
#3	“randomised” [Title/Abstract] OR “clinical trial” [Title/Abstract] OR “placebo” [Title/Abstract]
#4	#1 AND #2 AND #3

### Data Collection and Analysis

#### Selection of Studies

**Figure 1:**
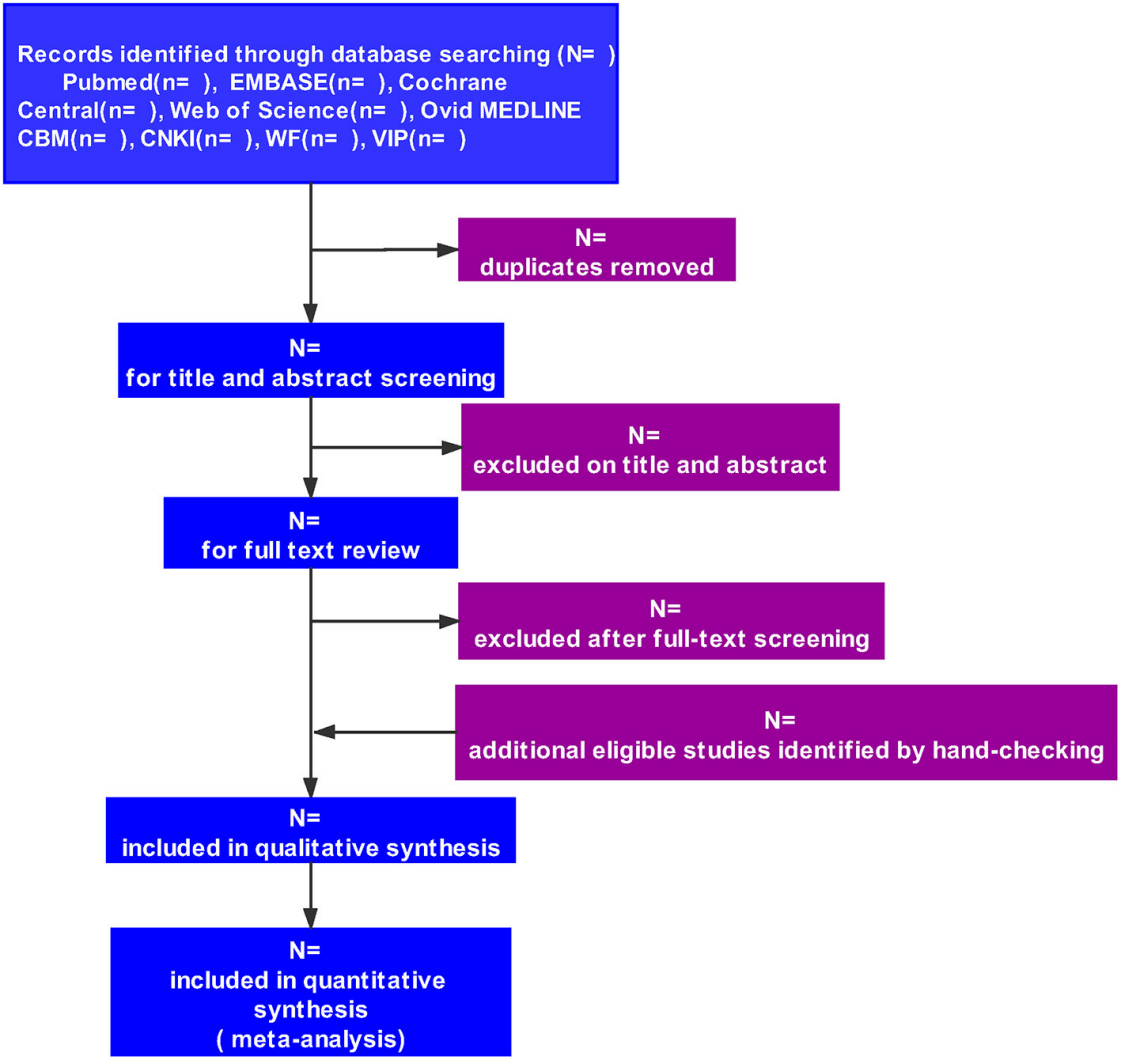
Flow diagram of study selection.

Two reviewers (YW and YX) will independently perform the study selection by assessing the titles and abstracts using Endnote software (V.X9.0) and applying the inclusion and exclusion criteria after removing duplicates. Full-text articles will be reviewed if necessary. Consensus will be achieved to resolve disagreements. The procedure for selecting the studies is shown in Figure 1.

#### Data Extraction and Management

Relevant data will be extracted by two reviewers (WY and LJ) independently using a standardised form, which includes the first author's name, date of publication, country, language, age, sex, sample size, duration of the disease, severity of the symptoms at baseline, follow-up, acupuncture (e.g., the specific acupuncture methods, number of sessions, duration, acupuncture points) and control interventions, outcome measures, results of the intervention, duration of follow-up assessments, and reported adverse effects. We will contact the corresponding author to retrieve additional information if necessary. Consensus will be achieved to resolve disagreements. Before proceeding with the review, consistency between the reviewers will be ensured by performing calibration exercises.

#### Assessment of the Risk of Bias

The quality of the included trials will be evaluated by two reviewers (YP and GD) using the Cochrane Collaboration tool. Seven domains (randomly generated sequence number, allocation concealment, blinding of participants, blinding of outcome assessment, incomplete outcome data, selective reporting, and other bias when required) will be assessed. For each aspect, the trial will be rated as having a high risk, a low risk, or an unclear risk of bias. A trial that is rated as having a high risk of bias in 1 or more aspects will be graded as “high risk”, while a trial with a low risk of bias in all aspects will be graded as “low risk”. If there is a low or unclear risk of bias for all main aspects, the trial will be rated as having “unclear risk”. The contact person or corresponding author will be contacted if basic information for the risk of bias assessment is missing. The rating results will be cross-checked, and discrepancies will be resolved through discussions and arbitration with a third author.

#### Measures of Treatment Effect

Estimations of the effect for dichotomous outcomes will be presented as risk ratios (RRs), while continuous outcomes will be expressed as weighted or standardised mean differences (SMDs). Ninety-five percent confidence intervals and two-sided *P* values will be calculated for each outcome.

#### Addressing Missing Data

We will contact authors by email to obtain missing data when feasible; otherwise, only the available data will be analysed.

#### Assessment of Heterogeneity

The χ^2^ test and I^2^ statistic will be applied to evaluate the heterogeneity of the studies. In addition, 95% CIs will be used to measure the reliability. Heterogeneity will be considered if *P* < 0.1 for the χ^2^ test or I^2^ > 50%.

#### Data Synthesis

RevMan software (V 5.3) will be used to perform the meta-analysis. Dichotomous and continuous data will be pooled using the Mantel–Haenszel method and the inverse variance method, respectively. A fixed-effects model will be used to combine the data when statistical heterogeneity is not considered, and a random-effects model will be used when statistical heterogeneity is considered. We will qualitatively synthesise the data when meta-analysis is not possible.

#### Subgroup Analysis and Investigation of the Heterogeneity

If statistical heterogeneity (*P* < 0.1 for the χ^2^ test or I^2^ > 50%) is observed, we will explore the clinical source(s) of the heterogeneity by subgroup analyses when sufficient data are available. Subgroup analyses will be based on the following: (1) the sex of the study population; (2) the age range of the study population: <40 years old, 40–60 years old, and older than 60 years old; and (3) the specific acupuncture methods (i.e., acupuncture, electroacupuncture, elongated needle acupuncture, internal heat-type acupuncture, intradermal needle-embedding therapy, warm acupuncture, fire needle acupuncture, auricular acupuncture, abdominal acupuncture, acupoint injection and floating needle therapy). A *P* < 0.1 for the χ^2^ test will be regarded as a statistically significant difference between subgroups.

#### Assessment of Publication Bias

Publication bias will be assessed using Egger's test and funnel plots.

#### Sensitivity Analysis

Publication bias will be assessed using Egger's test and funnel plots.

#### Evaluation of the Quality of Evidence

The quality of the evidence will be assessed by the Grading of Recommendations Assessment, Development, and Evaluation methodology (GRADE) and will be rated as high, moderate, low, or very low.

## Discussion

Shoulder pain is one of the most common musculoskeletal disorders among adults, with a lifetime incidence of up to 67% (42). A variety of conditions may contribute to shoulder pain. Instead of a single predisposing pathological state, multiple conditions may lead to the occurrence of shoulder pain. In clinical practise, it is often difficult to determine the contribution of different pathological conditions to shoulder pain, and the diagnosis of shoulder pain is usually determined by the condition with the most significant pathological change or clinical signs. Acupuncture, in a broad sense, refers to a variety of acupuncture techniques, including acupuncture, electroacupuncture, elongated needle acupuncture, internal heat-type acupuncture, intradermal needle embedding therapy, warm acupuncture, fire needle acupuncture, auricular acupuncture, abdominal acupuncture, acupoint injection and floating needle therapy. Green et al. (30) performed a systematic review on the effect of acupuncture on shoulder pain, but only acupuncture and electroacupuncture were included for evaluation, and little evidence was established to support or refute the use of acupuncture for shoulder pain. To comprehensively evaluate the efficacy of various acupuncture techniques in the treatment of shoulder pain, we will extensively include various acupuncture techniques. Hall et al. (31) performed a systematic review to assess the effect of trigger point dry needling in the shoulder region on upper extremity pain and dysfunction. The participants included in this review were patients with lateral elbow pain, simple myofascial pain, postoperative shoulder pain, hemiparetic shoulder pain, and nonspecific shoulder pain. The participants of our study will be patients with atraumatic shoulder conditions, including rotator cuff tears, subacromial impingement, osteoarthritis, adhesive capsulitis, tendinitis, tendinopathy, tenosynovitis, and bursitis. Dong et al. (43) performed a systematic review to comprehensively compare treatments for shoulder impingement syndrome. This review included 52 studies, and only 2 of these studies focused on acupuncture for shoulder impingement syndrome. Therefore, their conclusion on the comparison of the efficacy of acupuncture in the treatment of shoulder impingement syndrome and the efficacy of other methods is not strong. Ben-Arie et al. (32) performed a systematic review that showed very low-quality evidence that acupuncture has a superior effect on adhesive capsulitis when compared to other interventions. In the present review, we will extensively search the literature on the use of various acupuncture techniques for shoulder pain, and our study will include a variety of pathologies for shoulder pain, including subacromial impingement syndrome and adhesive capsulitis.

The nonsurgical management of shoulder pain includes physical therapy, nonsteroidal anti-inflammatory drugs, analgesic agents, and the local injection of steroids and/or local anaesthetics. As an important means of complementary and alternative medicine, acupuncture is widely used in treating musculoskeletal disorders, but the effects of acupuncture and other nonsurgical treatments are still inconclusive. The purpose of the present study is to systematically review and assess the effectiveness of a wide range of acupuncture techniques for atraumatic shoulder conditions.

## Ethics Statement

Since no primary data will be collected, ethical approval is not required for this systematic review. The findings of this systematic review will be published in a peer reviewed journal.

## Author Contributions

This study was conceived by YW, YX, and YP. YW, YX, YP, and GD drafted the manuscript. YP, SL, GD, and TL participated in the design of the data synthesis and analysis scheme. SL, GD, and TL reviewed and revised the manuscript. All authors have read and approved the publication of the protocol, contributed to the article, and approved the submitted version.

## Conflict of Interest

The authors declare that the research was conducted in the absence of any commercial or financial relationships that could be construed as a potential conflict of interest.

## Publisher's Note

All claims expressed in this article are solely those of the authors and do not necessarily represent those of their affiliated organizations, or those of the publisher, the editors and the reviewers. Any product that may be evaluated in this article, or claim that may be made by its manufacturer, is not guaranteed or endorsed by the publisher.
